# The Causes and Treatment of Œdema

**Published:** 1905-07-22

**Authors:** 


### THE CAUSES AND TREATMENT OF ŒDEMA.

Dr. J. Dixon Mann [1] argues that cardiac œdema is not directly the result of back pressure, but is chiefly due to changes in the vascular endothelium, which in turn depend on the imperfect supply of nutrient material and oxygen associated with defective circulation of the blood. Hence the vascular walls are unduly permeable and there is excessive transudation of fluid. The œdema of renal disease is less readily explained. The prejudiced condition of the walls of the blood-vessels, due to the presence of toxic bodies retained in the blood consequent on defective action of the kidneys, the retention of water from the same cause, and probably also the occurrence of hydræmic plethora are among the main factors. Of recent years attention has been paid to the relation between œdema and the sodium chloride of the blood and tissues. There is some reason to believe that the glomerular epithelium in renal disease keeps back a considerable amount of sodium chloride, and there is no doubt that with much sodium chloride in the tissues the amount of water there retained is relatively large. But the presumption thus suggested is not in entire harmony with the facts of clinical experience, and hence it cannot be said that the retention of sodium chloride is the exclusive cause of renal œdema. At the same time there is no doubt that a restriction in the dietetic supply of the salt in the œdema of Bright's disease tends to promote dispersion of the fluid.

In œdema dependent on cardiac disease the chief claim is for physiological rest for the heart. This finds an apt illustration in the fact that in many such cases the amount of urine passed during the night is in excess of the amount voided during the day—a reversal of the normal condition. The passive state of the patient during rest and sleep permits the cardiac energy to be devoted wholly to the nutrition of the tissues and to the transmission of an adequate blood-pressure to the kidney and other secretory organs. Often, however, rest alone is not sufficient to meet a state of defective compensation in cardiac disease. Of medicinal remedies digitalis occupies the first place, but, unfortunately, the pharmaceutical preparations of this drug are not constant in strength, as the percentage of active principle yielded by the plant in different years varies over a wide limit. Much of the ordinary tincture is, therefore, nearly or quite inert. More reliable preparations are Nativelle's granules of digitaline and a form of digitoxin known as "digalen." Alcohol may tide the heart over a crisis, but its action requires to be closely watched; in renal disease it is best avoided unless in exceptional circumstances. In œdema from renal incompetence the impairment of the glandular epithelium may neutralise the value of digitalis and other remedies which raise the blood-pressure; and the diuretic action of salines is, at best, uncertain. A number of diuretics of comparatively recent introduction are, however, of distinct value. Thus, while caffeine and theobromine each stimulate the heart and increase the activity of the kidney the action of the latter substance as a diuretic is much the more powerful. Theocine (now made synthetically) is still more active in this direction; the dose of this should not exceed 6 to 7 grains, or vomiting may be excited. Each of the three substances just mentioned, but especially theocine, increases both the fluids and solids of the urine, and hence theocine is particularly valuable in œdema accompanied by retention of sodium chloride. Both with it and the other xanthin derivatives diuresis may be delayed for 24 or more hours, and if the renal epithelium is profoundly disorganised there may be obvious failure in the action of the remedy. The combination of digitalis with one or other of these substances is often advantageous.

When diuretics fail to get rid of renal œdema the skin may be used as an avenue to excrete both water and solids. This may be effected by the use of the vapour bath or the hot-air bath. It is not wise to allow during the bath the free administration of water, as this, if swallowed, is added to the water already retained, and thus goes to neutralise the effective action of the bath. Cathartics, though of great service in Bright's disease, are of little value in reducing œdema. Dr. Dixon Mann also discusses the therapeutic relationship of food and drinks to œdema.

[1] Brit. Med. Jour., May 20, 1905.

